# Genome-wide analysis of KAP1, the 7SK snRNP complex, and RNA polymerase II

**DOI:** 10.1016/j.gdata.2016.01.019

**Published:** 2016-02-04

**Authors:** Ryan P. McNamara, Carlos Guzman, Jonathan E. Reeder, Iván D'Orso

**Affiliations:** a Department of Microbiology, The University of Texas Southwestern Medical Center, Dallas, TX 75390, USA; b The University of Texas Southwestern Medical Center, Dallas, TX 75390, USA

**Keywords:** P-TEFb, positive transcription elongation factor b, Pol II, RNA polymerase II, TSS, transcription start site, Bp, base pairs, KAP1, Kruppel-associated protein, Cdk9, cyclin-dependent kinase 9, CycT1, cyclin T1, MePCE, methyl phosphate capping enzyme, ChIP, chromatin immunoprecipitation, snRNP, small nuclear ribonucleoprotein, KEC, KAP1-7SK snRNP early elongation complex, Larp7, La-related protein 7, Hexim1, hexamethylene bis-acetamide inducible protein 1, qPCR, quantitative PCR, ChIP-seq, chromatin immunoprecipitation sequencing, GRO-seq, Global run on sequencing, P-TEFb/7SK snRNP, KAP1, RNA polymerase II, ChIP-seq, Transcription elongation

## Abstract

The transition of RNA polymerase II (Pol II) from transcription initiation into productive elongation in eukaryotic cells is regulated by the P-TEFb kinase, which phosphorylates the C-terminal domain of paused Pol II at promoter-proximal regions. Our recent study found that P-TEFb (in an inhibited state bound to the 7SK snRNP complex) interacts with the KAP1/TRIM28 transcriptional regulator, and that KAP1 and the 7SK snRNP co-occupy most gene promoters containing paused Pol II. Here we provide a detailed experimental description and analysis of the ChIP-seq datasets that have been deposited into Gene Expression Omnibus (GEO): GS72622, so that independent groups can replicate and expand upon these findings. We propose these datasets would provide valuable information for researchers studying mechanisms of transcriptional regulation including Pol II pausing and pause release.

**Table:** 

Specifications	
Organism/cell line/tissue	HCT116 colon carcinoma cell line (ATCC CCL-247)
Sex	Male
Sequencer or array type	Illumina
Data format	Raw and analyzed
Experimental factors	None
Experimental features	ChIP-seq of Pol II, KAP1, 7SK snRNP components (Cdk9, Hexim1, and Larp7), and chromatin signatures (H3K4me3, H3K4me1, and H3K27Ac) to identify target genes of KAP1 and the 7SK snRNP complex
Consent	Data are publicly available
Sample source location	American Type Culture Collection (ATCC), Manassas, VA, USA

## 1 Direct link to deposited data

Deposited data can be found at: http://www.ncbi.nlm.nih.gov/geo/query/ acc.cgi?acc = GSE72622.

## 2 Experimental design, materials and methods

### 2.1 Cell culture and reagents

The human colorectal HCT116 cell line (obtained from ATCC) was maintained (at a confluency not greater than 90%) in DMEM supplemented with 10% heat-inactivated FBS and 1 × penicillin/streptomycin at 37 °C with 5% CO_2_.

### 2.2 Crosslinking and sonication of cells for ChIP assay

Low passage (< 5) HCT116 cells were grown in T150 cm^2^ flasks and removed from the plate using 0.05% trypsin–EDTA and 1 × PBS, and spun down at 1000 *g* at 4 °C for 5 min. Excess trypsin/PBS was aspirated and the cell pellet was resuspended to a density of 1 × 10^7^ cells/mL in PBS. Methanol-free formaldehyde (16% stock) was added dropwise to a 0.5% final concentration and gently nutated at room temperature for 10 min. To ensure that no cell clumps formed, the cell suspension was pipetted up and down throughout several times during the crosslinking step. Crosslinking was quenched through the dropwise addition of glycine to a final concentration of 0.125 M and gently nutated at room temperature for 10 min. The crosslinked cell suspension was then spun down at 800 *g* at 4 °C for 5 min. The cell pellet was washed twice (spun down at 1000 *g* at 4 °C for 5 min) in cold PBS to eliminate excess formaldehyde and glycine.

The cell pellet was then resuspended in cold Farnham lysis buffer (5 mM piperazine-N,N′-bis(2-ethanesulfonic acid) (PIPES) pH 8.0, 85 mM KCl, 1 mM PMSF, 1 × EDTA-free protease inhibitor, 0.5% NP-40) to a final cell density of 1 × 10^7^ cells/mL and dounce homogenized (10 strokes) in a 1 mL dounce homogenizer using a loose pestle (Wheaton). The cell suspension was then nutated at 4 °C for 30 min and spun down at 1000 *g* at 4 °C for 10 min. These combined steps are to separate cytosolic components from the nucleus. The supernatant (cytosol) was discarded and the pellet (nuclei) was resuspended in cold Szak's RIPA buffer (50 mM Tris–HCl pH 8.0, 150 mM NaCl, 2.5 mM EDTA, 1% NP-40, 0.1% SDS, 0.5% deoxycholic acid, 1 × EDTA-free protease inhibitor, 1 mM PMSF) to a final density of 2.5 × 10^7^ cells/mL and dounce homogenized (10 strokes) in a 1 mL dounce homogenizer using a tight pestle (Wheaton). The nuclear lysate was then transferred to thin-walled 1.5 mL TPX tubes (Diagenode) for a final volume of 250 μL per tube (this is a critical limit that must be respected). Samples were then sonicated until DNA was fragmented to an average distribution of about 200–300 bp using a water bath Bioruptor (Diagenode) for a total of 45 cycles (30 s on and 30 s off). To further ensure sonication efficiency, we allowed a 20 min cool-down of the water bath after every 20 cycles. To verify that DNA was fragmented to the appropriate size (200–300 bp average distribution), a fraction of lysate was reverse crosslinked, the DNA was purified and run on a 1.5% agarose gel stained with ethidium bromide alongside a 100 bp ladder at 5 V/cm.

### 2.3 ChIP assay

Dynabeads Protein G (10004D, Life Technologies) (70 μL of slurry per ChIP) were equilibrated with RIPA buffer. 4 μg of each antibody diluted in 500 μL of RIPA (see McNamara et al. [1]) was conjugated to beads at room temperature for 1 h. Antibody-coated beads were then blocked with 0.3 mg/mL BSA diluted in RIPA at 4 °C for 1 h. Then, the sonicated nuclear lysate (from point 2.2) was added to the blocked antibody-coated beads and nutated at 4 °C for 2 h. Given the low levels of ChIP recovery for some of the 7SK snRNP components, we optimized the amount of sonicated cell nuclei used per ChIP assay as follows: 2.5 × 10^7^ cell nuclei for Pol II, H3K4me3, H3K4me1, and H3K27ac; and 5.0 × 10^7^ cell nuclei for KAP1, Cdk9, Hexim1, and Larp7. Unbound nuclear lysate was discarded and beads were washed twice with the following cold buffers:

1. RIPA buffer (see above),
2. Low salt buffer (1% NP-40, 20 mM Tris–HCl pH 8.0, 150 mM NaCl, 0.1% SDS, 0.5% deoxycholic acid, 2.5 mM EDTA, 1 mM PMSF),
3. High salt buffer (1% NP-40, 20 mM Tris–HCl pH 8.0, 500 mM NaCl, 0.1% SDS, 0.5% deoxycholic acid, 2.5 mM EDTA, 1 mM PMSF),
4. LiCl buffer (1% NP-40, 20 mM Tris HCl pH 8.0, 250 mM LiCl, 0.1% SDS, 0.5% deoxycholic acid, 2.5 mM EDTA, 1 mM PMSF),
5. 1 × Tris–HCl EDTA buffer (TE).

After washing, complexes were eluted off the beads using elution buffer (1% SDS and 100 mM NaHCO_3_) at 65 °C for 30 min, with intermittent vortexing. Protein-DNA complexes were decrosslinked using decrosslinking buffer (50 mM Tris–HCl pH 6.8, 500 mM NaCl, 5 mM EDTA, 0.5 mg/mL Proteinase K) at 60 °C for 4 h. DNA was then purified using the Zymo ChIP DNA Clean and Concentrator Kit (Zymo Research).

### 2.4 ChIP DNA quality control

All ChIP DNA samples were quantified for concentration using the Qubit® 2.0 Fluorometer (Thermo Fisher Scientific). A small fraction of input and ChIP DNA was used to test for enrichment at the *NFKBIA* promoter (a Pol II paused gene) prior to library preparation and sequencing. The non-Pol II paused gene *GREB1* was used as a negative control [1], [2]. Given that our ChIP DNA samples showed enrichment at the Pol II paused *NFKBIA* promoter-proximal region (but not at the *GREB1* negative control gene), we submitted our samples for high-throughput sequencing.

### 2.5 ChIP DNA library preparation and sequencing

10–20 ng DNA (Input or ChIP from Point 2.3) were submitted to the McDermott Center Sequencing Core at UT Southwestern Medical Center for library preparation and high-throughput sequencing using the NextSeq500 (Illumina). Samples were end repaired, 3′-end adenylated and barcoded with multiplex adapters (Applied Biosystems). After purification with Ampure XP beads (Beckman Coulter), samples were PCR amplified for 15 cycles, size selected with Ampure XP beads, and quantified on the Agilent 2100 BioAnalyzer. Emulsion PCR was then performed and beads enriched on the EZ-Bead system. Each sample yielded 2.5–5.0 × 10^7^ total reads of 50 nt, depending on the sample. For each sample, ~ 70–80% of unique reads mapped to the human genome (GRCh37/hg19).

## 3 Data analysis and results

**Fig. 1:**
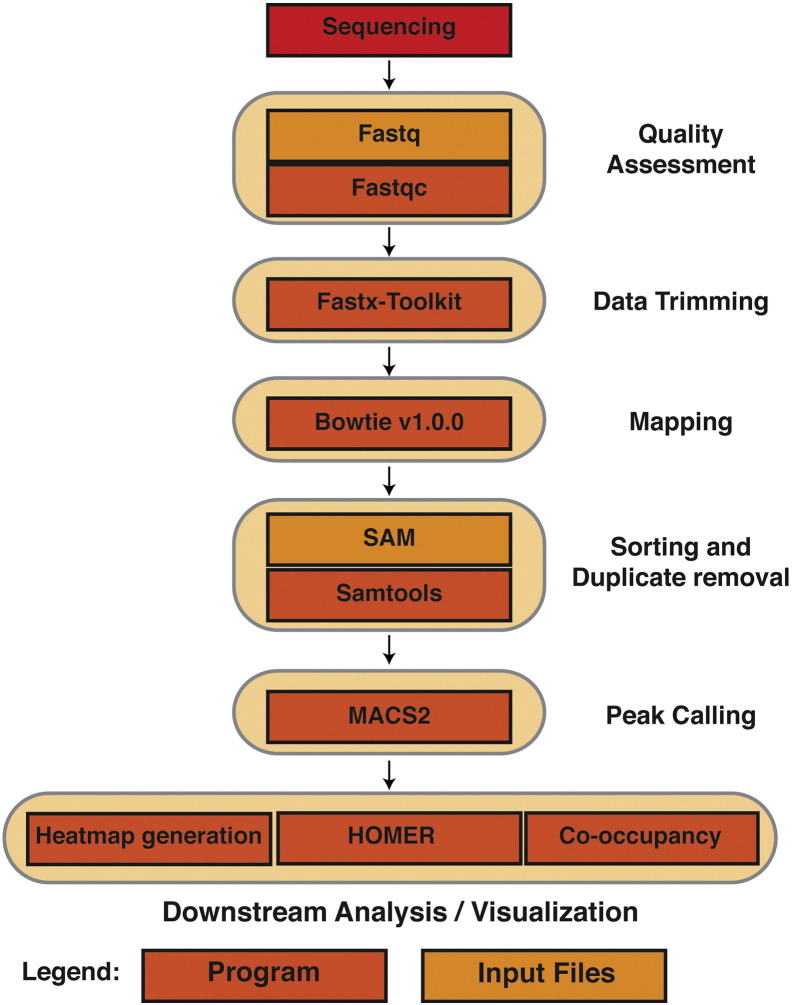
Flowchart showing the pipeline used for ChIP-seq data analysis. See text for details.

Fig. 1 shows a detailed flowchart description of the steps/tools used for data analysis.

### 3.1 Computational analysis and data transformation

All scripting was performed using Python 2.7.6. All ChIP-seq binding events were loaded into a custom MySQL database to allow for efficient comparisons of multiple factors' binding loci. Fastq files obtained from the sequencing core were first checked for quality using Fastqc, and then trimmed of any adapters, barcodes, and low-quality sequences using the Fastx toolkit (Fig. 1). For mapping and sorting, the ChIP-seq output was aligned to the reference human genome (GRCh37/hg19) using Bowtie v1.0.0 [3], allowing 1 mismatch (− v 1). Sequences aligning to multiple locations in the reference genome were discarded. − S was specified to deliver .sam output, which was then sorted and converted to binary (.bam) using Samtools [4]. For ChIP-seq peak calling, the .sam files for all ChIP-seq marks were analyzed for binding events using peak calling in the MACS2 software [5]. When determining peaks, duplicate read alignments were discarded to avoid amplification artifacts from the sequencing PCR. All ChIP-seq files were normalized to input sequencing files.

### 3.2 ChIP-seq data visualization

MACS2 was run in the callpeak mode with the –*B* and –*SPMR* flags set to generate signal pileup tracks in bedGraph format on a per million reads basis. This allows for direct comparison between individual tracks, regardless of any differences in the sequencing depth. The bedgraph files were converted to BigWig files using a custom script based on the University of California at Santa Cruz (UCSC) tool ‘bedGraphToBigWig’. The pileup tracks (.BigWig files) were visualized using Integrative Genomics Viewer (IGV) [6]. The normalized pileup tracks also served as the input for many data visualizations as noted below.

### 3.3 Generation of ChIP-seq heatmaps

HOMER [7] annotatePeaks.pl was used to generate density matrices using normalized bedGraph pileup tracks of ChIP-seq marks as input. The –*hist* flag was set with a parameter of 50 bp bins and the –*ghist* flag was also set to generate a density matrix. After density matrixes were generated they were fed into Cluster 3.0 to generate a .cdt file. A custom script was then used to rank the resulting .cdt file in order of decreasing density. We took the Pol II matrix and sorted it by decreasing total density and then clustered all other ChIP-seq matrices to the same sort order as Pol II. The resulting .cdt matrices were then fed into Java TreeView 3.0 [8] to generate visual heatmaps.

### 3.4 Generation of ChIP-seq metagene plots

HOMER [7] annotatePeaks.pl was used to generate metagene plots based on the average profile of a series of ChIP-seq marks in normalized bedGraph pileup tracks. The –*hist* flag was set using 50 bp bins for all metagene profiles. Plots were centered on the TSS and extending the indicated window (e.g. ± 1 kb (− size 2000)).

### 3.5 GRO-seq analysis

We analyzed the nascent RNA-sequencing data (GRO-seq) generated by the Espinosa laboratory in the HCT116 cell line [9] to compare transcription status with levels of Pol II and KAP1-7SK snRNP occupancy at promoter-proximal regions. We ranked RefSeq genes located in the positive strand in the human genome based on decreasing sense transcript levels surrounding the TSS. We then sorted the ChIP-seq datasets of Pol II, KAP1, Cdk9, Hexim1, and Larp7 based on ranked GRO-seq data. GRO-seq Fastq files [9] were downloaded from the ftp server of the European Bioinformatics Institute (EBI) (ftp://ftp.sra.ebi.ac.uk/vol1/fastq/) and then aligned using Bowtie v1.1.2 allowing for up to two mismatches (− n 2) resulting in a sam output file (− S). Sam files were then converted into sorted bam using Samtools v1.3 and further converted into strand specific genome coverages using bedtools (genomeCoverageBed). Entire documented analysis and parameters can be found on our Github (https://github.com/Dorsolab/GD_GenomicsData_Analysis).

### 3.6 Genome-wide factor distribution and co-occupancy analysis

To characterize the genome-wide distribution of the 7SK snRNP, we employed an unbiased ChIP-seq approach to localize the individual components: Larp7 (constitutively bound to the 3′ of 7SK RNA), Hexim1 (7SK RNA-binding protein that tethers P-TEFb to the 7SK snRNP), and Cdk9 (the kinase subunit of P-TEFb) [10]. We interrogated the locations of these three components to identify with high-confidence genomic domains marked by the 7SK snRNP (as opposed to the detection of individual subunits not assembled on the snRNP). We also examined the distribution of KAP1 to determine if it co-occupies genomic domains with the 7SK snRNP, possibly forming a KAP1-7SK snRNP complex [1].

**Fig. 2:**
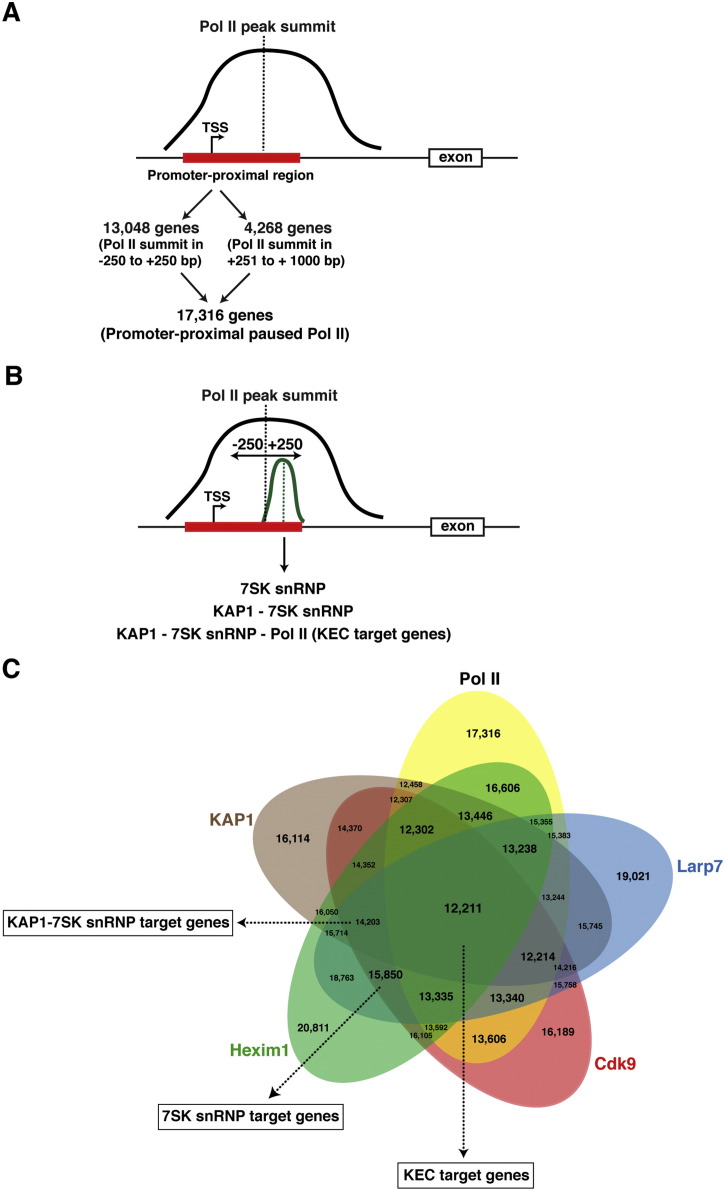
Strategy to identify 7SK snRNP, KAP1-7SK snRNP and KEC (KAP1-7SK snRNP early Elongation Complex) target genes in the human genome. (A) Search for Pol II peak summit in the promoter-proximal region. (B) Search for factor co-occupancy in the − 250 to + 250 bp region from the Pol II peak summit at promoter-proximal regions. (C) Factor co-occupancy analysis as explained in panel (B).

To identify KAP1 and 7SK snRNP target genes we searched for KAP1, Hexim1, Larp7, and Cdk9 narrow peaks (as defined by MACS) at promoter-proximal regions of genes listed in the UCSC RefSeq database (30,180 unique TSS). For the occupancy analysis, we defined a broader promoter-proximal region window than others have used (− 250 to + 1000 bp from the TSS). These thresholds were selected because we found 13,048 genes having a Pol II summit using a narrow window (− 250 to + 250 bp from the TSS) and 4268 additional genes containing a Pol II summit, in addition to an H3K4me3 peak, at + 250 to + 1000 bp from the TSS (Fig. 2A). It is unclear, however, whether this increase in genes containing paused Pol II in the broader window is due to inaccurate TSS annotations and/or that in some genes Pol II pauses further downstream from the + 100 site, as the Pol II metagene analysis indicates (see below). Therefore, the broader window (− 250 to + 1000 bp from the TSS) was used for the co-occupancy analysis, to more completely capture genes that have all the hallmarks of paused Pol II, even though the pausing may appear downstream of more restrictive bounds of a currently annotated TSS.

To identify genes targeted by KAP1, the 7SK snRNP, and Pol II (referred to as KEC target genes for KAP1-7SK snRNP early Elongation Complex), we first searched for Pol II summit locations in the broad promoter-proximal region (− 250 to + 1000 bp from TSS) and analyzed for co-occupancy of all KAP1-7SK snRNP components within a window of − 250 to + 250 bp from the Pol II summit (narrow peak as determined by MACS) (Fig. 2B). This filter guarantees that the factors are within a region where Pol II may realistically be considered paused, and that they are all within a distance to interact (directly or indirectly) with one another. We found 17,316 genes containing promoter-proximal paused Pol II, 15,850 7SK snRNP target genes, 14,203 KAP1-7SK snRNP target genes, and 12,211 KEC target genes (Fig. 2B and C). This last number represents a large fraction of all RefSeq genes (40.5% of all annotated genes and 70.5% of genes containing paused Pol II), which prompted us to conclude that most active/paused genes contain the KAP1-7SK snRNP complex in the promoter-proximal region. Clearly, our combined experimental/computational approach is underestimating the number of genes targeted by the 7SK snRNP, as can be seen by the lack of one of the three 7SK snRNP components (probably due to technical difficulties with ChIP-seq and/or the peak calling algorithm) (Fig. 2C).

### 3.7 A statistical analysis defines that KAP1, the 7SK snRNP, and Pol II are enriched at promoter-proximal regions of active/paused genes

We found that the majority of KAP1 and components of the 7SK snRNP complex mapped to promoter and promoter-proximal regions of most genes containing paused Pol II [1]. Given our proposed role of KAP1-7SK snRNP in promoting transcriptional elongation, we examined the overlap of KAP1-7SK snRNP with Pol II outside the promoter-proximal region, and calculated p-values for co-occupancy using the hypergeometric density distribution with the phyper function in the R stats package. For this analysis, we collapsed the entire genome into 250 bp bins, removing any region that corresponded to − 250 to + 1000 bp from the TSS. Using this window, we found that paused Pol II is present at the promoter-proximal region of 17,316 genes, and that the KAP1-7SK snRNP is present at the promoter proximal-region of 14,203 genes, which represents an intersection of 12,211 genes. This overlap is significant at a p-value < 2.2204^− 16^ (machine zero), as calculated using a hypergeometric density distribution. In addition, we found paused Pol II at the promoter-proximal region of genes (n = 551) devoid of KAP1-7SK snRNP, indicating significant de-enrichment of genes having paused Pol II but no (or very low levels) KAP1-7SK snRNP at these sites (p-value < 2.2204^− 16^). Together, this indicates that Pol II and the KAP1-7SK snRNP significantly co-localize at promoter-proximal regions.

### 3.8 KAP1 and the 7SK snRNP complex are deposited downstream of paused Pol II

**Fig. 3:**
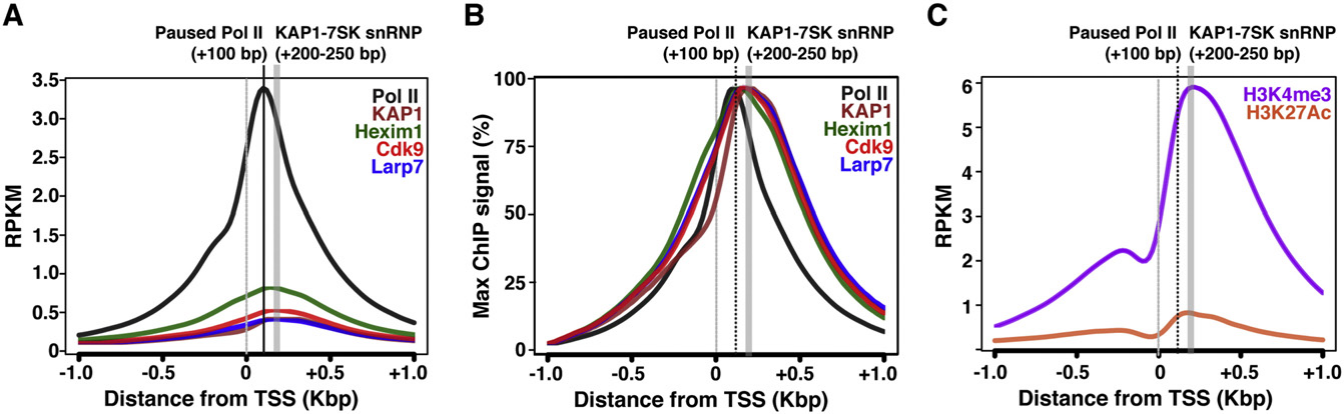
Mapping the location of KAP1 and the 7SK snRNP complex relative to the Pol II pause site. (A) Metagene analysis showing factor distribution surrounding the TSS with accurate signal strengths. RPKM are Reads Per Kilobase of Transcript per Million mapped reads. (B) Metagene analysis showing factor distribution surrounding the TSS where the RPKM signal was normalized to the maximum (Max) ChIP signal and expressed as percentage (%) for ease of comparison. (C) Metagene analysis showing the distribution of the chromatin signatures (H3K4me3 and H3K27Ac) surrounding the TSS.

Given the high frequency of Pol II and KAP1-7SK snRNP co-occupancy at promoter-proximal regions, we generated metagene plots to define their average relative proximity to the TSS. The Pol II metagene reveals that the average pause site occurs at about + 100 bp from the TSS (Fig. 3A and B), shifted slightly downstream of the paused site originally identified in other Pol II Chip-chip or ChIP-seq datasets [11], [12]. These differences in Pol II pause site might be attributable to the different species (humans or *Drosophila*) used in the experimental approaches, number of genes being averaged depending on the species analyzed, and/or mean signal densities. Higher resolution approaches like GRO-seq [13] and PRO-seq [14] concluded that Pol II pauses, on average, at about + 50 bp from the TSS thus showing that our Pol II mean densities calculations might be accurate reflections of the broad distribution, and much lower resolution, obtained with ChIP-seq.

Interestingly, the metagene profiles for components of the KAP1-7SK snRNP complex peaked downstream of the Pol II pause site, mirroring the active chromatin signatures H3K4me3 and H3K27Ac (Fig. 3C), indicating that the average KAP1-7SK snRNP peak falls about 100–150 bp downstream of the paused Pol II summit. Additionally, we also noticed a fairly broad distribution pattern (+ 200–250 bp from the TSS) for KAP1 and components of the 7SK snRNP complex (Fig. 3A and B). This might be attributable to the different placement of KAP1-7SK snRNP complexes at distinct groups of genes respective to the TSS. Further experimental and computational work is needed to define groups of genes with different positioning of the KAP1-7SK snRNP complex respective to the TSS and its functional relationship to chromatin architecture and transcriptional status.

### 3.9 Relationship between transcription activity, Pol II pausing, and recruitment of the KAP1-7SK snRNP to promoter-proximal regions

**Fig. 4:**
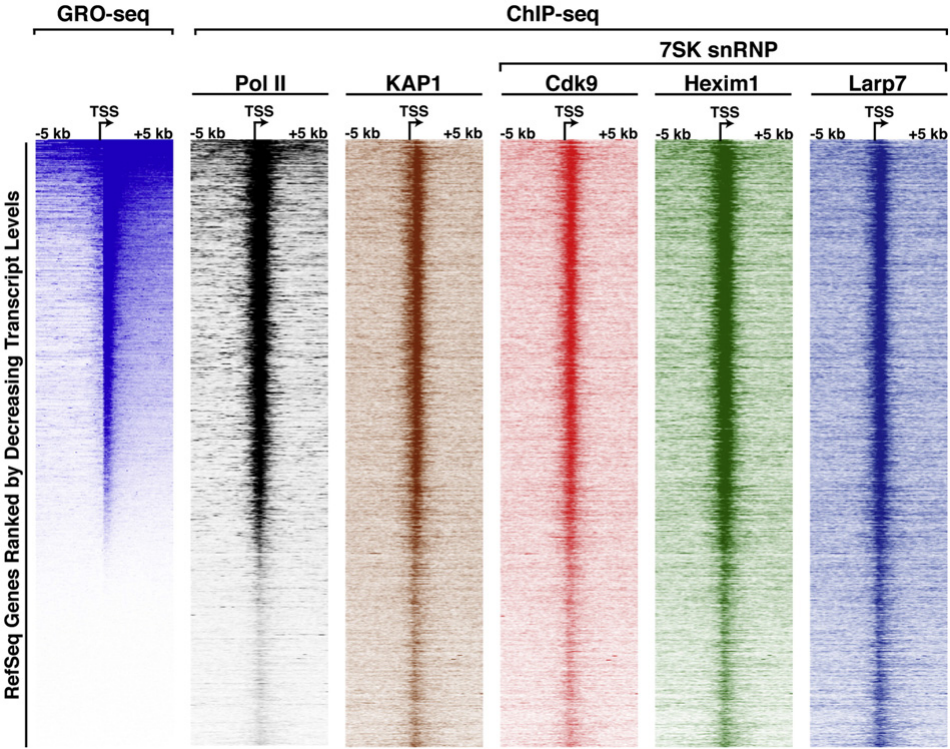
Relationship between transcription activity, Pol II pausing, and recruitment of the KAP1-7SK snRNP to promoter-proximal regions. GRO-seq shows only positive strand information.

To define the relationship between Pol II pausing, KAP1-7SK snRNP deposition at promoter-proximal regions and transcriptional levels, we re-analyzed the nascent RNA-sequencing data (GRO-seq) generated by the Espinosa laboratory [9]. After ranking RefSeq genes located in the positive strand in the human genome based on decreasing levels of transcription surrounding the TSS, we observed that the distribution and levels of sense strand transcription mirror the occupancy levels of paused Pol II and KAP1-7SK snRNP components (Fig. 4). Together, the analysis indicates that paused Pol II and KAP1-7SK snRNP co-occupy promoter-proximal regions of transcriptionally active/paused genes and that there is a positive correlation between levels of paused Pol II and KAP1-7SK snRNP at these sites.

## 4 Discussion

We described here an experimental approach to determine the occupancy of KAP1, the 7SK snRNP and Pol II in the human genome. Using this collection of ChIP-seq datasets we demonstrated that the transcriptional regulator KAP1 and the 7SK snRNP complex co-occupy (with high frequency) promoter-proximal regions containing paused Pol II. We propose that this collection will provide valuable information for researchers studying mechanisms of transcriptional regulation, particularly Pol II pausing and pause release. Certainly, future experimental and computational approaches are needed to further define if KAP1 recruits the 7SK snRNP to most promoter-proximal regions containing paused Pol II and whether the KAP1-7SK snRNP complex regulates pause release genome-wide. Our main work has closely examined annotated transcripts but not divergent transcripts or non-coding RNAs. Thus, it remains to be elucidated whether these unique non-coding RNA species are also targeted by KAP1 and the 7SK snRNP or whether they have unique regulatory strategies. Moreover, given that enhancers play an important role in controlling gene activation from promoters and because the 7SK snRNP appears to be recruited to these genomic sites [15], [16], future studies are needed to further define the role of KAP1-7SK snRNP in controlling transcription from enhancers during development and cell differentiation. Our work lays the foundation for these future studies.

## Conflict of interest

The authors declare that there are no conflicts of interests.
